# Flexible thin-film thermal sensor for estimating thermal transport properties designed for biomaterial applications

**DOI:** 10.1038/s41598-025-03304-0

**Published:** 2025-05-28

**Authors:** Takahiro Okabe, Ayumi Shiroto, Yuto Hiyama, Katsuhisa Taguchi

**Affiliations:** 1 Graduate School of Science and Technology, Hirosaki University, Hirosaki, Japan; 2 SEMITEC Corporation, Sumida, Japan

**Keywords:** Heat transfer, Thermal conductivity, Volumetric heat capacity, Thermal contact resistance, Inverse analysis, Heat conduction, Mechanical engineering, Biomedical engineering

## Abstract

This study develops a flexible thin-film thermal sensor for estimating the key thermal transport properties of biomaterials, namely thermal conductivity, volumetric heat capacity, and thermal contact resistance. The sensor included a circular thin-film heater and three thin-film NTC thermistors mounted on a polyimide substrate, enabling simultaneous temperature measurements in regions of heat concentration and diffusion. These measurements were used in an inverse analysis based on a three-dimensional heat conduction model. To account for the variability in contact conditions, the thermal contact resistance between the sensor and sample surface was included as an unknown parameter. A trust-region reflective algorithm was used for robust minimization of the objective function. Validation was conducted using standard materials with known properties and porcine fat as a biological sample. The results showed excellent agreement between the measured and simulated temperature profiles. The estimated values for thermal conductivity and volumetric heat capacity closely matched reference values, and the thermal contact resistance was consistently in the order of 10^−4^ (m^2^ K)/W, likely owing to surface roughness. These results demonstrate the effectiveness of the proposed sensor and the inverse analysis method in enabling noninvasive and accurate evaluation of thermal transport properties in soft biological tissues.

**Supplementary Information:**

The online version contains supplementary material available at 10.1038/s41598-025-03304-0.

## Introduction

Characterization of the thermophysical behavior of biological tissues has long been central to bioheat transfer research. A comprehensive investigation of tissue thermal transport properties, such as thermal conductivity and diffusivity, is vital for advancing and refining diagnostic^1–8^ and therapeutic^9–13^ approaches using heat transfer phenomena in medical applications. The correlation between tissue properties and their physical or pathological states allows for a more precise prediction of therapeutic outcomes and the monitoring of disease progression. Therefore, the accurate measurement of these properties in biomaterials is essential for the development of novel medical technologies and for ensuring their safety and efficacy.

The thermal transport properties of solid materials are typically measured using various commercially available methods, the choice of which depends on the material and specific conditions. The laser flash method^14,15^, which is widely used for measuring thermal diffusivity, provides high accuracy but requires precise specimen preparation and surface coating, particularly for transparent samples. The transient plane source method is effective for poorly conducting materials, in which a sensor is clamped between two polished samples to minimize the thermal contact resistance. For noninvasive measurements, the point contact method^16–18^ is used, although thermal contact remains a critical concern as a heated probe is pressed onto the surface of the sample.

Although these methods offer advantages and are applicable to a wide range of materials, they are generally unsuitable for soft materials, such as biomaterials. For example, the laser flash method requires precise specimen preparation, which is challenging to achieve using soft materials. Similarly, the transient plane source method struggles to clamp soft specimens without causing deformation. Moreover, the point contact method, which assumes point contact between the sensor and sample, faces challenges because pressing a bead probe onto a soft sample alters the contact area and thermal contact resistance.

Several researchers have attempted to develop effective techniques to measure the thermophysical properties of biological tissues. Among these, the self-heated thermistor method is recognized as one of the most suitable techniques for measuring the thermal conductivity of biological tissues, owing to its speed and the simplicity of the equipment required^19–21^. However, this is an invasive technique that can cause tissue trauma owing to probe insertion. Consequently, numerous researchers have concluded that, despite its advantages, clinical experimentation with actual human tissue cannot be conducted owing to ethical issues.

To address the issues associated with invasive techniques, numerous researchers have developed noninvasive methods to measure the thermophysical properties of biological tissues and minimize tissue trauma^22–25^. These methods generally estimate properties such as thermal conductivity and diffusivity via inverse analysis^26,27^, which involves capturing the temperature response on the skin surface when subjected to heating or cooling. By comparing this temperature response with theoretical models, the underlying thermal properties can be inferred. However, current noninvasive approaches often rely on complex systems and are restricted in their capacity to assess arbitrary body regions or facilitate continuous monitoring outside laboratory environments.

In this study, we propose the development of a flexible thin-film thermal sensor designed to enable rapid and quantitative estimation of the thermal conductivity and volumetric heat capacity of biomaterials. The sensor has a unique and minimal configuration that allows for simultaneous temperature measurements in two key areas, where heat is concentrated and it diffuses, within a single module, leading to more accurate parameter estimation. Additionally, to address variations in contact conditions during the measurements, the thermal contact resistance between the sensor and sample surface was included as an estimation parameter in the inverse analysis. This new method aims to overcome the limitations of existing methods, such as the requirement for complex equipment and inability to measure arbitrary body regions and consider the state of contact, making it suitable for continuous monitoring in real-world clinical settings. To demonstrate the feasibility and effectiveness of the proposed method, we conducted experiments using well-characterized phantom materials and evaluated the accuracy and robustness of the parameter estimation.

## Method

### Experimental setup

**Fig. 1:**
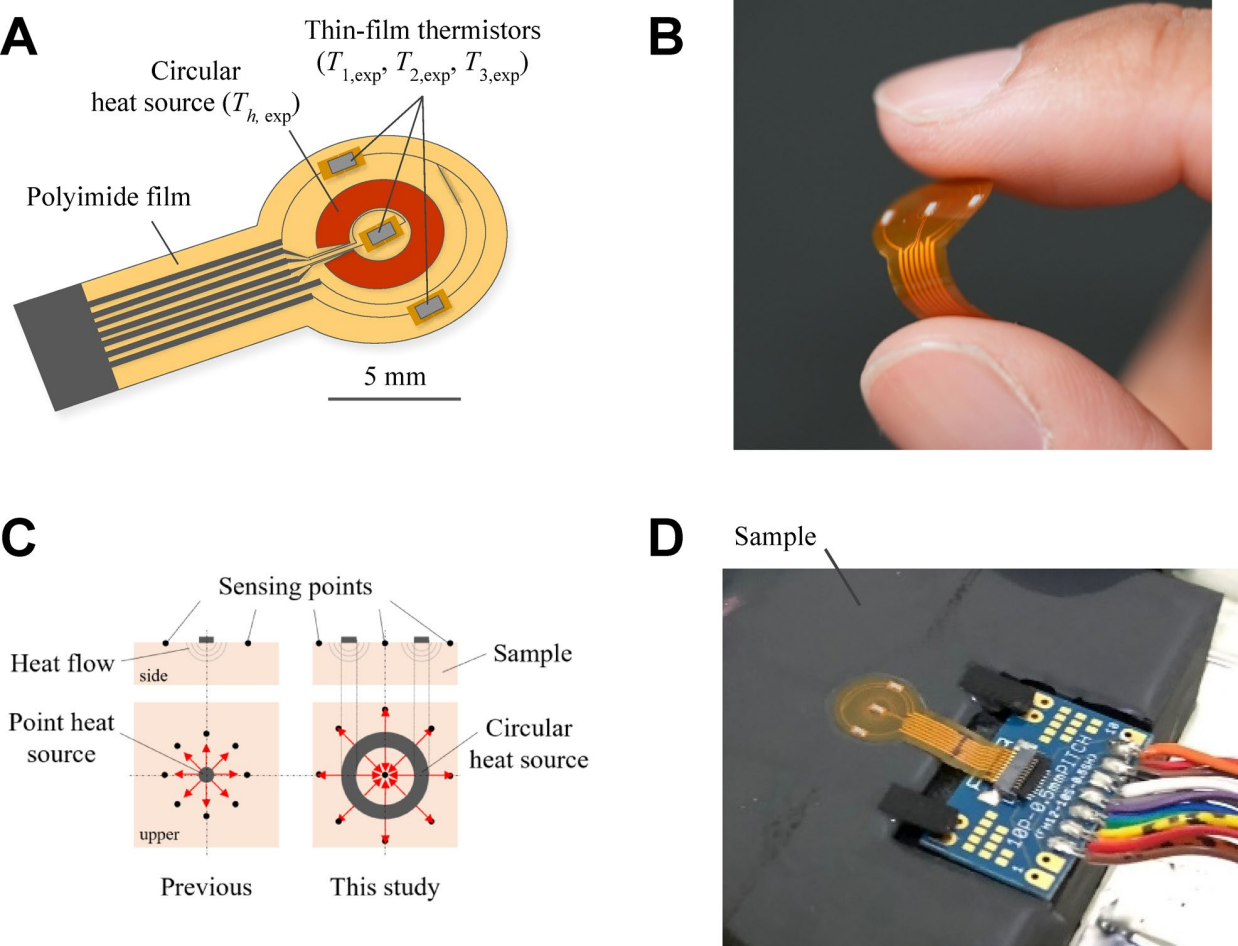
A flexible and thin-film thermal sensor: (**A**) schematic of the sensor module, (**B**) photograph demonstrating the sensor’s flexibility, (**C**) comparison of heat flow between previous and current studies, and (**D**) photograph showing the measurement in progress without the sensor’s protective cover.

Figure 1A,B show the flexible thin-film thermal sensor designed to estimate the thermal transport properties of biomaterials. The sensor consisted of a circular thin-film heater (inner diameter: 5 mm, outer diameter: 10 mm) and three thin-film NTC thermistors (1.0 mm × 0.50 mm × 0.15 mm, 103FT1005, SEMITEC Co.) mounted on a 50 μm thick polyimide substrate. The thin-film NTC thermistors used in this study differed from conventional thermistors in terms of their ultracompact size, fast response time, and high heat resistance, offering reliable performance in dynamic thermal measurements. A key feature of this sensor is the circular configuration of the heat source, which allows thermal stimulation not at a single point, as commonly used in previous studies, but across an annular region. This configuration enabled the simultaneous acquisition of temperature data in both the heat concentration and diffusion regions, as shown in Fig. 1C. Consequently, the sensor enhances sensitivity and reproducibility, thereby improving the accuracy of the inverse parameter estimation. This is particularly beneficial for noninvasive measurements, where depth-resolved thermal information is inherently limited.

The thermistors and circular heater were independently connected to a digital multimeter (7352A, ADCMT) to monitor the resistance during operation. The heater was powered by a DC power supply (KX-S-210L, TAKASAGO), and all the devices were interfaced with a personal computer using LabVIEW (National Instruments) for synchronized control and data acquisition. Prior to the experiments, calibration was performed to establish the relationship between the electrical resistance and temperature of both the heater and the thermistors. The uncertainty in temperature measurement was evaluated by combining repeatability and instrument resolution and is discussed in Supplementary Section S1.

### Experimental procedure

The following experimental procedure was used for all tests on both the standard materials and porcine fat. As standard materials with thermophysical properties similar to those of biological tissues, acrylic resin, silicone rubber, polyethylene, and agar-gelled water were prepared. Prior to measurement, the sample surfaces were gently cleaned to ensure stable thermal contact and allowed to reach thermal equilibrium to establish consistent initial conditions. The flexible thermal sensor was then attached using thermally conductive double-sided tape, as shown in Fig. 1D.

To minimize heat loss due to natural convection from the sensor surface, a custom-made protective cover was placed over the flexible sensor during the experiment. An air layer was interposed between the sensor and the cover, forming a thermally insulating gap that suppresses convective disturbances near the sensing area. The thickness of this layer was sufficiently small (≈ 1 mm) to ensure that the Rayleigh number remained below the threshold for natural convection, thereby maintaining stable and uniform thermal conditions around the sensor.

A constant power of 117 mW was supplied to the circular thin-film heater, and the temperature responses at the heater and three NTC thermistors were recorded for 5 s immediately after the heating began. Data were sampled at intervals of 0.067 s using a digital multimeter. This interval was determined by the measurement range and operation mode of the multimeters, along with the LabVIEW-based control system, and was selected to ensure sufficient temporal resolution for capturing the early-stage transient behavior.

All experiments were conducted at 21.9 ± 0.6 °C under still air conditions. To ensure thermal equilibrium between successive measurements, the sensor was left to rest for 3–5 min until the temperature returned to the baseline. Each measurement was repeated five times at the same location to confirm repeatability. All acquired temperature profiles were analyzed using the inverse analysis method developed in this study.

### Mathematical model

**Fig. 2:**
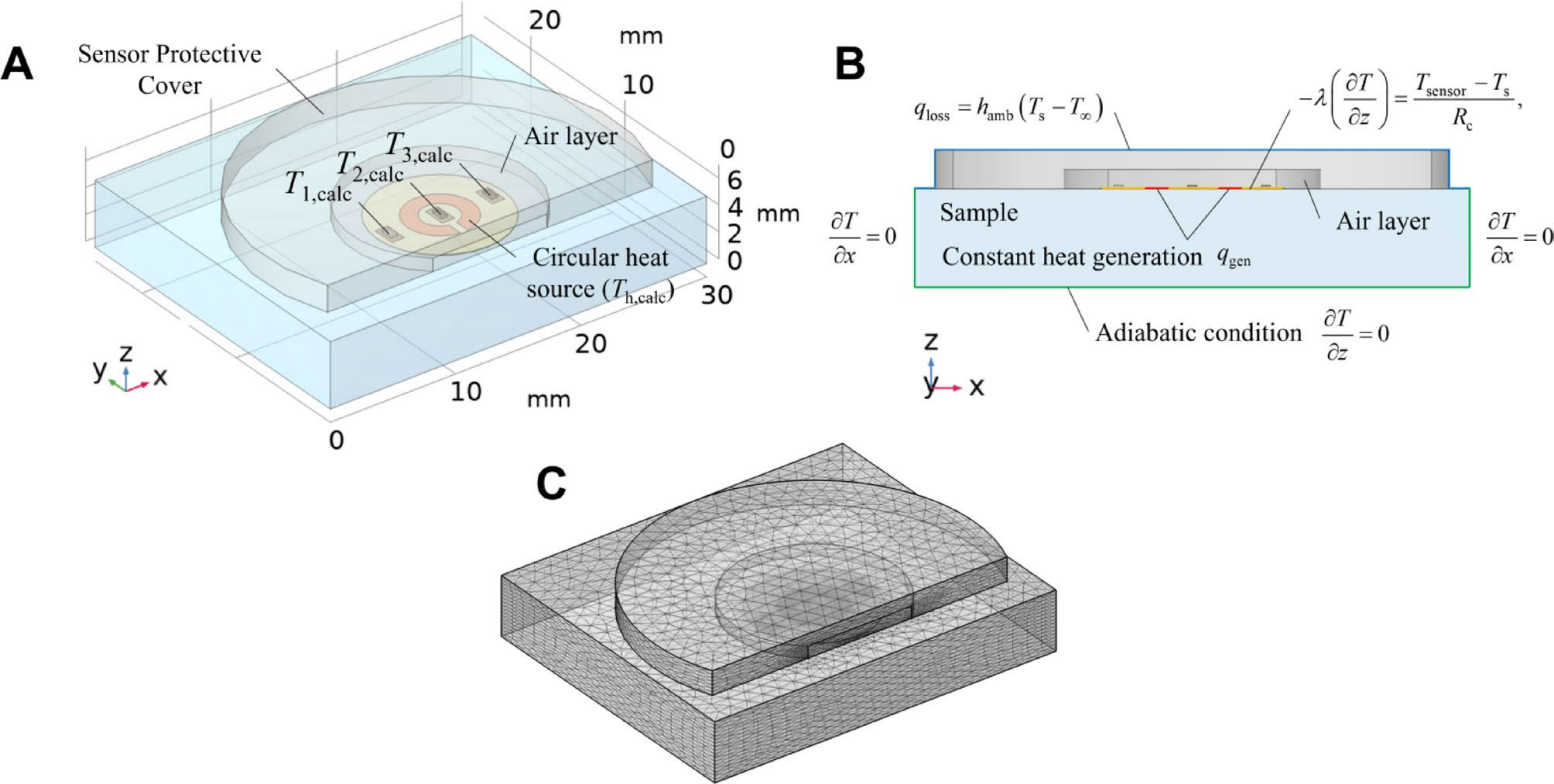
(**A**) 3-D heat conduction model for calculating the temperature responses, (**B**) side view model with boundary conditions, and (**C**) computational mesh.

Figure 2A,B show the 3D heat conduction model consisting of four components: a flexible thermal sensor, a sensor-protective cover, an air layer between the sensor and cover, and a sample. The model calculated the transient heat conduction during the heating of the sample for 5 s. The temperature responses of the two thermistors, *T*_calc,1_, *T*_calc,2_, and heater *T*_calc,h_ were extracted for inverse reconstruction analysis. The governing equations are as follows:

1 $$\rho c\frac{\partial T}{{\partial t}} = \lambda \nabla^{2} T + q_{{{\text{gen}}}} ,$$

where *ρ*, *c*, *t*, *λ*, and *q*_gen_ denote density [kg/m^3^], specific heat [J/(kg·K)], time [s], thermal conductivity [W/(m·K)], and heat generation rate [W/m^3^], respectively.

To account for the thermal contact resistance between the sensor and sample surface, the boundary conditions at the contact interface were modeled by incorporating *R*_c_, which is defined as:

2 $$- \lambda \left( {\frac{\partial T}{{\partial z}}} \right) = \frac{{T_{{{\text{sensor}}}} - T_{{\text{s}}} }}{{R_{{\text{c}}} }},$$

where $\left( {{{\partial T} \mathord{\left/ {\vphantom {{\partial T} {\partial z}}} \right. \kern-0pt} {\partial z}}} \right)$ denotes the temperature gradient at the interface [K/m], *T*_sensor_ and *T*_s_ denote the temperatures at the sensor and sample surfaces [K], respectively, and *R*_c_ denotes the thermal contact resistance [(m^2^·K)/W]. This relationship helps to account for the heat transfer across the interface and is used as an additional parameter in the inverse analysis to improve the estimation accuracy of the thermal transport properties.

The surfaces of the sample and sensor-protective cover were exposed to ambient air during the experiment, resulting in heat loss to the atmosphere. As shown in Fig. 2B, the heat loss was described by the convective boundary condition as follows:

3 $$q_{{{\text{loss}}}} = h_{{{\text{amb}}}} \left( {T_{{\text{s}}} - T_{\infty } } \right),$$

where *h*_amb_ represents the heat transfer coefficient, *T*_s_ denotes the temperature at the sample and sensor cover surfaces exposed to the ambient environment, and *T*_∞_ denotes the ambient temperature, which is 21.9 ± 0.6 °C based on the conditions. The heat transfer coefficient was set to 10 W/(m^2^·K), to account for the effects of natural convection and radiation from the surface. As confirmed by parametric testing, small variations in the surface heat transfer coefficient due to ambient fluctuations had negligible impact on the measurement results, as the cover effectively stabilized the heat loss pathway. The other boundary condition is adiabatic, as follows:

4 $$\frac{\partial T}{{\partial x}} = 0,\,\,\,\,\,\frac{\partial T}{{\partial y}} = 0,\,\,\,\,\,\frac{\partial T}{{\partial z}} = 0.$$

The initial temperature distribution was considered as the ambient temperature *T*_∞_ for all of the components. The numerical solutions of Eq. (1) were obtained using the finite element software COMSOL Multiphysics ver. 6.2. A typical mesh generated for the computational domain consisted of 469,657 elements (Fig. 2C).

### Inverse reconstruction

**Fig. 3:**
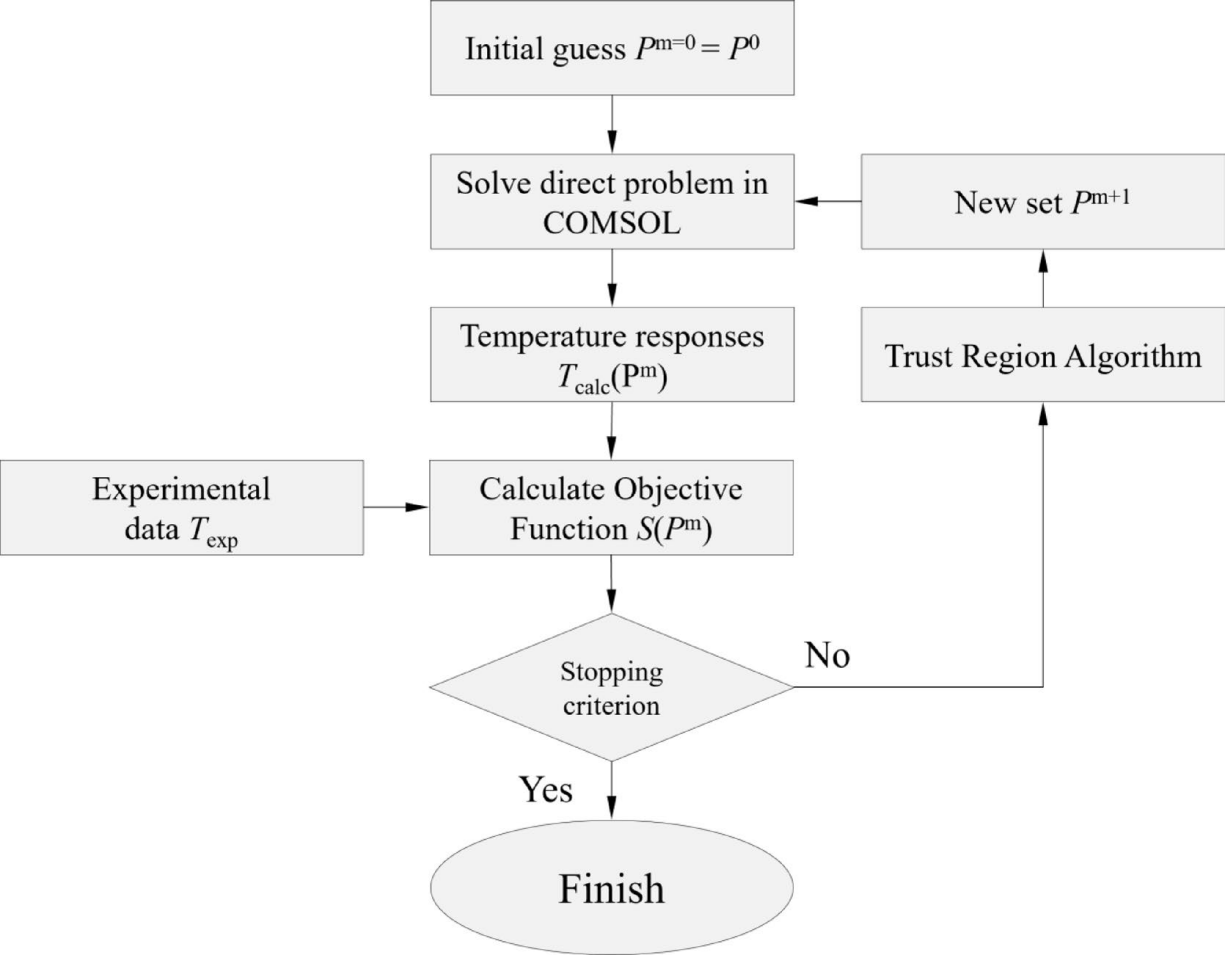
Flowchart of the inverse reconstruction algorithm for parameter estimation.

The inverse reconstruction of the thermal transport properties based on the measured temperature data involves several steps. A flowchart of the inverse analysis is shown in Fig. 3. The algorithm begins by solving a direct problem using an initial guess for the set of unknown parameters *λ*, *ρc*, and *R*_c_, which are stored in the vector **P**^0^, as follows:

5 $${\mathbf{P}}^{0} = \left[ {\begin{array}{*{20}c} {\lambda_{{{\text{s}}0}} } \\ {\left( {\rho c} \right)_{{{\text{s}}0}} } \\ {R_{{{\text{c0}}}} } \\ \end{array} } \right],$$

Here, the initial guesses for *λ*_s0_, (*ρc*)_s0_, and *R*_c0_ were 0.1 W/(m·K), 1.0 MJ/(m^3^·K), and 0.5 × 10^−4^ (m^2^·K)/W, respectively.

The algorithm proceeds from the initial estimates to the iteratively improved estimates of the unknown parameters using a trust-region reflective method implemented in the optimization toolbox of MATLAB. This algorithm is a subspace trust-region approach based on the interior-reflective Newton method, as described in previous studies^29,30^. Each iteration approximately solved a large linear system using the preconditioned conjugate gradient method. The iterative process continues until the difference between the measured and calculated temperatures falls below the tolerance thresholds defined in MATLAB, including the function tolerance, step size tolerance, and gradient tolerance. These parameters ensure convergence within a predefined accuracy range.

We selected the trust-region reflective algorithm because it supports bound-constrained optimization and demonstrated superior convergence behavior in preliminary tests. Although alternative solvers, such as the Levenberg–Marquardt algorithm, are also available in MATLAB, they exhibited greater sensitivity to initial guesses and occasionally failed to converge to physically meaningful solutions. In contrast, the trust-region reflective algorithm consistently yielded stable and robust estimates across a range of initial guesses, justifying its use in this study.

The set of unknown parameters at the *m-*th iteration is represented by the vector **P**^*m*^, as follows:

6 $${\mathbf{P}}^{m} = \left[ {\begin{array}{*{20}c} {\lambda_{{\text{s}}}^{m} } \\ {\left( {\rho c} \right)_{{\text{s}}}^{m} } \\ {R_{{\text{c}}}^{m} } \\ \end{array} } \right],$$

The temperature responses calculated from the three-dimensional heat conduction model correspond to a specific set of parameters **P**^*m*^ are expressed as *T*_*i*,calc_(*t*, **P**^*m*^), where **P**^*m*^ includes the thermal transport parameters to be estimated. The values of the target parameters were determined by searching for the optimal set that minimized the difference between the calculated temperatures (**T**_1,calc_, **T**_2,calc_, and **T**_h,calc_) and experimentally measured temperatures (**T**_1,exp_, **T**_2,exp_, and **T**_h,exp_). The temperature vectors are defined as follows:

7 $${\mathbf{T}}_{{{1,}\,{\text{calc}}}} \left( {{\mathbf{P}}^{m} } \right) = \left[ {\begin{array}{*{20}c} {T_{{1,\,{\text{calc}}}} (t_{1} ,{\mathbf{P}}^{m} )} \\ {T_{{1,\,{\text{calc}}}} (t_{2} ,{\mathbf{P}}^{m} )} \\ \vdots \\ {T_{{1,\,{\text{calc}}}} (t_{n} ,{\mathbf{P}}^{m} )} \\ \end{array} } \right],\,\,\,\,\,\,{\mathbf{T}}_{{{2,}\,{\text{calc}}}} \left( {{\mathbf{P}}^{m} } \right) = \left[ {\begin{array}{*{20}c} {T_{{2,\,{\text{calc}}}} (t_{1} ,{\mathbf{P}}^{m} )} \\ {T_{{2,\,{\text{calc}}}} (t_{2} ,{\mathbf{P}}^{m} )} \\ \vdots \\ {T_{{h,\,{\text{calc}}}} (t_{n} ,{\mathbf{P}}^{m} )} \\ \end{array} } \right],\,\,\,\,\,\,{\mathbf{T}}_{{{\text{h,}}\,{\text{calc}}}} \left( {{\mathbf{P}}^{m} } \right) = \left[ {\begin{array}{*{20}c} {T_{{h,\,{\text{calc}}}} (t_{1} ,{\mathbf{P}}^{m} )} \\ {T_{{h,\,{\text{calc}}}} (t_{2} ,{\mathbf{P}}^{m} )} \\ \vdots \\ {T_{{h,\,{\text{calc}}}} (t_{n} ,{\mathbf{P}}^{m} )} \\ \end{array} } \right],$$

8 $${\mathbf{T}}_{{{1,}\,\exp }} = \left[ {\begin{array}{*{20}c} {T_{1,\,\exp } (t_{1} )} \\ {T_{1,\,\exp } (t_{2} )} \\ \vdots \\ {T_{1,\,\exp } (t_{n} )} \\ \end{array} } \right],\,\,\,\,\,\,{\mathbf{T}}_{{{2,}\,\exp }} = \left[ {\begin{array}{*{20}c} {T_{2,\,\exp } (t_{1} )} \\ {T_{2,\,\exp } (t_{2} )} \\ \vdots \\ {T_{2,\,\exp } (t_{n} )} \\ \end{array} } \right],\,\,\,\,\,\,{\mathbf{T}}_{{{\text{h,}}\,\exp }} = \left[ {\begin{array}{*{20}c} {T_{{{\text{h}},\,\exp }} (t_{1} )} \\ {T_{{{\text{h}},\,\exp }} (t_{2} )} \\ \vdots \\ {T_{{{\text{h}},\,\exp }} (t_{n} )} \\ \end{array} } \right],$$

where *n* denotes the number of measurements recorded over time. The objective function *S*(**P**^*m*^) is defined as the sum of the squares of the differences between the measured and calculated temperatures at three locations (*T*_1_, *T*_2_, and *T*_h_):

9 $$S({\mathbf{P}}^{m} ) = {\mathbf{r}}\left( {{\mathbf{P}}^{m} } \right)^{T} {\mathbf{r}}\left( {{\mathbf{P}}^{m} } \right),$$

10 $${\mathbf{r}}\left( {{\mathbf{P}}^{m} } \right) = \left[ {\begin{array}{*{20}c} {{\mathbf{T}}_{{{1,}\,{\text{calc}}}} \left( {{\mathbf{P}}^{m} } \right) - {\mathbf{T}}_{{\text{1,exp}}} } \\ {{\mathbf{T}}_{{{2,}\,{\text{calc}}}} \left( {{\mathbf{P}}^{m} } \right) - {\mathbf{T}}_{{\text{2,exp}}} } \\ {{\mathbf{T}}_{{{\text{h,}}\,{\text{calc}}}} \left( {{\mathbf{P}}^{m} } \right) - {\mathbf{T}}_{{\text{h,exp}}} } \\ \end{array} } \right] = \left[ {\begin{array}{*{20}c} {\begin{array}{*{20}c} {T_{{1,\,{\text{calc}}}} (t_{1} ,{\mathbf{P}}^{m} ) - T_{1,\exp } (t_{1} )} \\ {T_{{1,\,{\text{calc}}}} (t_{2} ,{\mathbf{P}}^{m} ) - T_{1,\exp } (t_{2} )} \\ \vdots \\ {T_{{1,\,{\text{calc}}}} (t_{n} ,{\mathbf{P}}^{m} ) - T_{1,\exp } (t_{n} )} \\ \end{array} } \\ {\begin{array}{*{20}c} {T_{{2,\,{\text{calc}}}} (t_{1} ,{\mathbf{P}}^{m} ) - T_{2,\exp } (t_{1} )} \\ {T_{{2,\,{\text{calc}}}} (t_{2} ,{\mathbf{P}}^{m} ) - T_{2,\exp } (t_{2} )} \\ \vdots \\ {T_{{h,\,{\text{calc}}}} (t_{n} ,{\mathbf{P}}^{m} ) - T_{h,\exp } (t_{n} )} \\ \end{array} } \\ {\begin{array}{*{20}c} {T_{{h,\,{\text{calc}}}} (t_{1} ,{\mathbf{P}}^{m} ) - T_{h,\exp } (t_{1} )} \\ {T_{{h,\,{\text{calc}}}} (t_{2} ,{\mathbf{P}}^{m} ) - T_{h,\exp } (t_{2} )} \\ \vdots \\ {T_{{h,\,{\text{calc}}}} (t_{n} ,{\mathbf{P}}^{m} ) - T_{h,\exp } (t_{n} )} \\ \end{array} } \\ \end{array} } \right],$$

MATLAB code using the optimization toolbox was developed to solve the inverse problem. During the optimization process, the temperature distributions corresponding to each set of input parameters were computed using COMSOL Multiphysics (ver. 6.2), a commercial finite element analysis software. These parameters were iteratively updated using an inverse reconstruction algorithm implemented in MATLAB. To enable the seamless communication and control of COMSOL using MATLAB, LiveLink was used for the MATLAB interface.

## Results

### Temperature responses

**Fig. 4:**
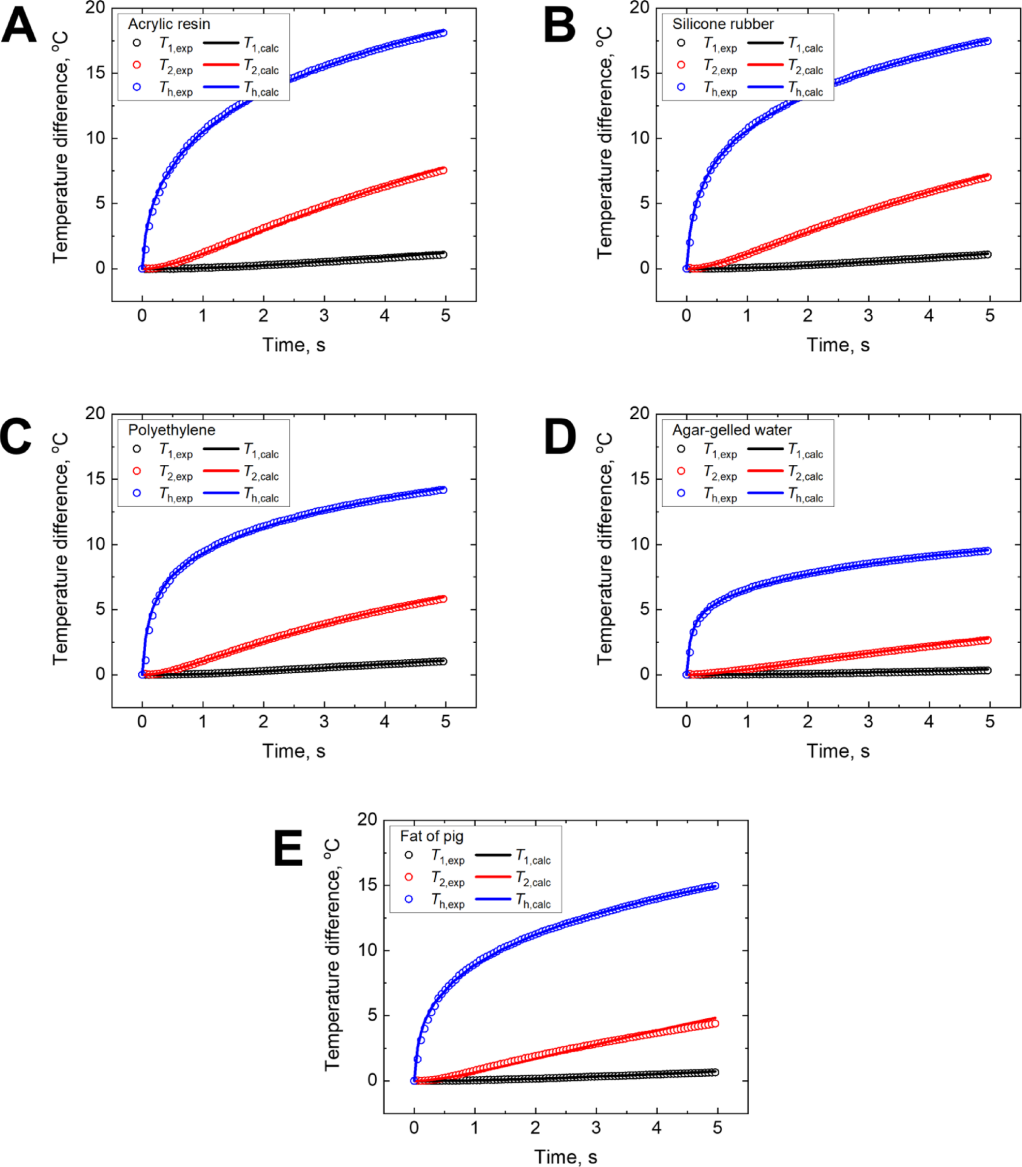
Comparison between measured and calculated temperature responses (*T*_1_, *T*_2_, and *T*_h_) for each sample after inverse analysis: (**A**) acrylic resin, (**B**) silicone rubber, (**C**) polyethylene, (**D**) agar-gelled water, and (**E**) fat of pig.

Figure 4 compares the experimental and calculated temperature responses of thermistors *T*_1_ and *T*_2_ and heater *T*_h_ for each sample. As designed, the circular heater supplied constant thermal power, resulting in heat accumulation near the inner region and outward radial diffusion owing to its unique geometry. Consequently, the temperatures at *T*_1_ and *T*_2_ differed, leading to distinct thermal behaviors between the heater and two thermistors. This spatial temperature difference played a crucial role in reducing the likelihood of local minima during the parameter estimation process and enhanced the robustness of the inverse analysis. This approach is particularly effective for solving inverse problems when the depth-resolved temperature information in biological tissues is difficult to obtain noninvasively.

Markedly different transient temperature increases were observed across the samples, reflecting variations in their thermophysical properties and thermal contact resistances. Samples with lower thermal diffusivities, such as acrylic resin and silicone rubber, exhibited greater temperature increases, whereas those with higher thermal diffusivities, such as agar-gelled water, exhibited moderate responses. The calculated temperature responses obtained via inverse analysis were in good agreement with the experimental measurements, thereby supporting the validity of the method.

To evaluate the quality of the acquired signals, the signal-to-noise ratio (SNR) was examined. The measured temperature rises at the sensor locations ranged from approximately 0.5 K to over 18 K, while the expanded uncertainty (*k* = 2) was 0.016 K (*T*_1_), 0.027 K (*T*_2_), and 0.12 K (*T*_h_), as determined from repeatability and resolution (Supplementary Section S1, Table S1). These results indicate that the SNR typically exceeds 10, and in many cases reaches values substantially higher, supporting the fidelity and reliability of the temperature signals used in the inverse analysis.

To better visualize the spatial development of heat transfer from the heater into the sample, the temperature field obtained from the numerical simulation is shown in Fig. S2 of the Supplementary Section S4. These visualizations present the lateral and vertical diffusion of heat over time, helping to interpret the timing and magnitude of the sensor responses described above.

**Fig. 5:**
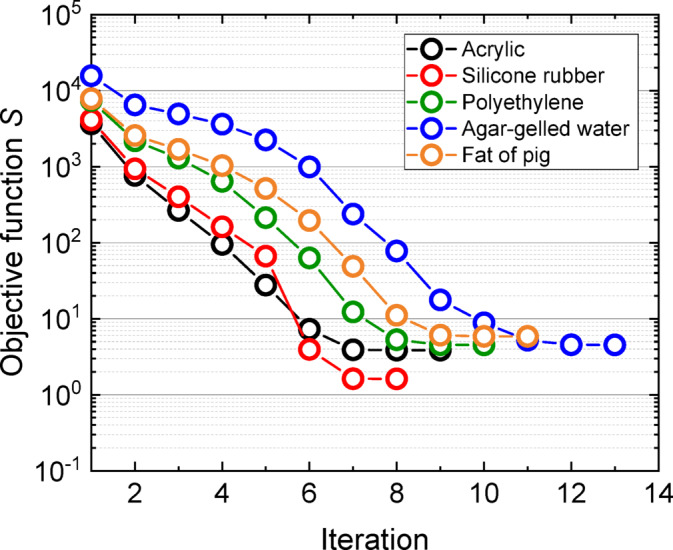
An example of the evolution of the objective function versus the number of iterations for each sample.

Figure 5 shows the variation in the objective function during the iterative process for each sample. The optimization was terminated once the objective function reached a predefined threshold. Convergence was typically achieved within 8 to 13 iterations, starting from arbitrary initial guesses: *λ*_s0_: 0.1 W/(m·K), (*ρc*)_s0_: 1.0 MJ/(m^3^·K), and* R*_c0_: 0.5 × 10^−4^ (m^2^·K)/W). This number of iterations was within a reasonable range, indicating that the algorithm was appropriately constructed to ensure convergence stability and computational efficiency. These results highlight the capability of the proposed inverse analysis approach to minimize the objective function robustly and efficiently, even for samples with various thermal properties, thereby demonstrating the general applicability and reliability of the method.

### Comparison between estimated and reference values

**Fig. 6:**
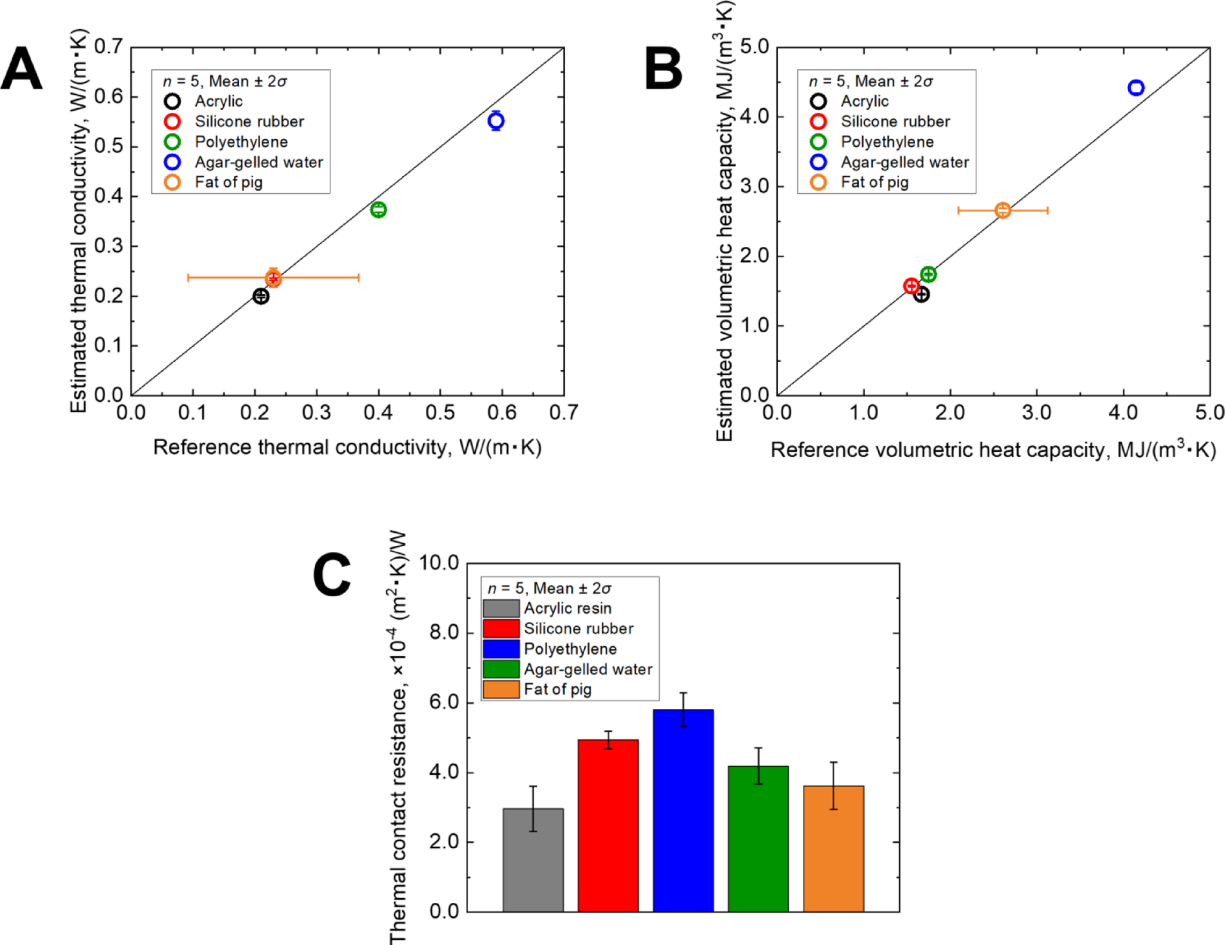
Comparison between estimated and reference values: (**A**) thermal conductivity, (**B**) volumetric heat capacity, and (**C**) thermal contact resistance.

The estimated thermal conductivity (*λ*), volumetric heat capacity (*ρc*), and thermal contact resistance (*R*_c_), obtained via inverse analysis for each sample, are shown in Fig. 6 and Table 1. Because the thermophysical properties of pig fat vary significantly based on its condition and composition, the horizontal error bars in the figure and the values in the table represent the ranges reported in previous studies^27^. Each measurement was repeated five times, yielding consistent and reproducible estimates for all the parameters.

**Table 1:** Estimated and reference values of thermal conductivity, volumetric heat capacity, and thermal contact resistance (*n* = 5)^28^.

	Thermal conductivity *λ* [W/(m·K)]		Volumetric heat capacity *ρc* [MJ/(m^3^·K)]		Thermal contact resistance *R*_c_ [× 10^−4^ (m^2^·K)/W]
	Estimated	Reference	Estimated	Reference	Estimated
Acrylic resin	0.200 ± 0.002	0.21	1.457 ± 0.0075	1.67	2.96 ± 0.65
Silicone rubber	0.234 ± 0.002	0.23	1.574 ± 0.0099	1.55	4.94 ± 0.25
Polyethylene	0.374 ± 0.005	0.40	1.741 ± 0.012	1.75	5.80 ± 0.48
Agar-gelled water	0.552 ± 0.019	0.59	4.418 ± 0.075	4.20	4.19 ± 0.52
Fat of pig	0.237 ± 0.019	0.094–0.37	2.659 ± 0.030	2.1–3.1	3.62 ± 0.68

Estimated results are presented as mean ± 2*σ*.

The average thermal conductivity values across the five replicates differed from the reference values by 4.8% for acrylic resin, 1.7% for silicone rubber, 6.5% for polyethylene, and 6.4% for agar-gelled water. The estimated results for pig fat, although variable owing to sample-specific conditions, were comparable to those reported in previous studies.

However, the estimated thermal contact resistance (*R*_c_) ranged from 2.96 × 10^−4^ to 5.80 × 10^−4^ (m^2^·K)/W, depending on the sample. This variation is attributed to differences in the surface conditions and contact pressure. However, because a thermally conductive double-sided adhesive tape was used between the sensor and sample to stabilize the thermal contact, the *R*_c_ values remained within the same order of magnitude across all samples. Notably, the estimated *R*_c_ values were of the same order as those reported for interfaces between the internal components in electronic devices, where the contact pressure was relatively low. This suggests that the values obtained in this study were reasonable and consistent with previously reported data (on the order of × 10^−4^ (m^2^·K)/W)^31^.

The estimated *R*_c_ comprised both the thermal resistance of the thermally conductive tape and that of the interposed air layer. Based on catalog specifications, the thermal conductivity and thickness of the tape were assumed to be 1.5 W/(m·K) and 40 μm, respectively, resulting in a tape thermal resistance of 2.67 × 10^−5^ (m^2^·K)/W. This value is one order of magnitude smaller than the total estimated *R*_c_. Accordingly, if the total thermal contact resistance *R*_c_ is primarily attributed to the air layer between the sensor and sample, a simple one-dimensional model, as expressed by Eq. (11), yields an equivalent air layer thickness ranging from 6.91 to 14.2 μm. This result is consistent with the expected microscale separation at such interfaces.

11 $$L_{{{\text{air}}}} = \lambda_{{{\text{air}}}} R_{{\text{c}}} ,$$

where *L*_air_ represents the equivalent thickness of the air layer interposed between the sensor and sample and *λ*_air_ represents the thermal conductivity of the air.

The standard deviation of the estimated values (2*σ*) also showed favorable ranges of 0.92–8.06% for thermal conductivity and 0.51–1.06% for volumetric heat capacity, confirming the high reproducibility and accuracy of the inverse analysis. By contrast, thermal contact resistance exhibited considerably larger variation, with a standard deviation ranging from 4.98 to 22.1%. This pronounced variability reflects the sensitivity of the thermal contact resistance to subtle differences in surface conditions and contact pressure and demonstrates the capability of the proposed method to detect and quantify such variations. Given that the other parameters showed good agreement with the reference values, this result underscores the importance and effectiveness of incorporating thermal contact resistance as a parameter in the inverse analysis based on contact temperature measurements.

To further evaluate the robustness of the estimated parameters against experimental uncertainty, an uncertainty analysis based on the Monte Carlo Method (MCM) was performed. Thirty synthetic datasets were generated by adding normally distributed noise, derived from the combined standard uncertainties of each sensor, to the original temperature signals. The resulting statistical distributions of *λ*, *ρc*, and *R*_c_ confirmed the stability and reliability of the proposed method. The details of this analysis are provided in Supplementary Section S2.

### Sensitivity analysis

To further examine the identifiability of each parameter, dimensionless sensitivity coefficient *X*_s_ was evaluated over time using forward simulations based on the estimated parameters. *X*_s_ is defined as:

12 $$X_{{\text{s}}} = \frac{p}{T} \cdot \frac{\partial T}{{\partial p}},$$

where *p* is the thermal transport parameters of interest (i.e., thermal conductivity *λ*, volumetric heat capacity *ρc*, or thermal contact resistance *R*_c_), and *T* is the temperature response.

**Fig. 7:**
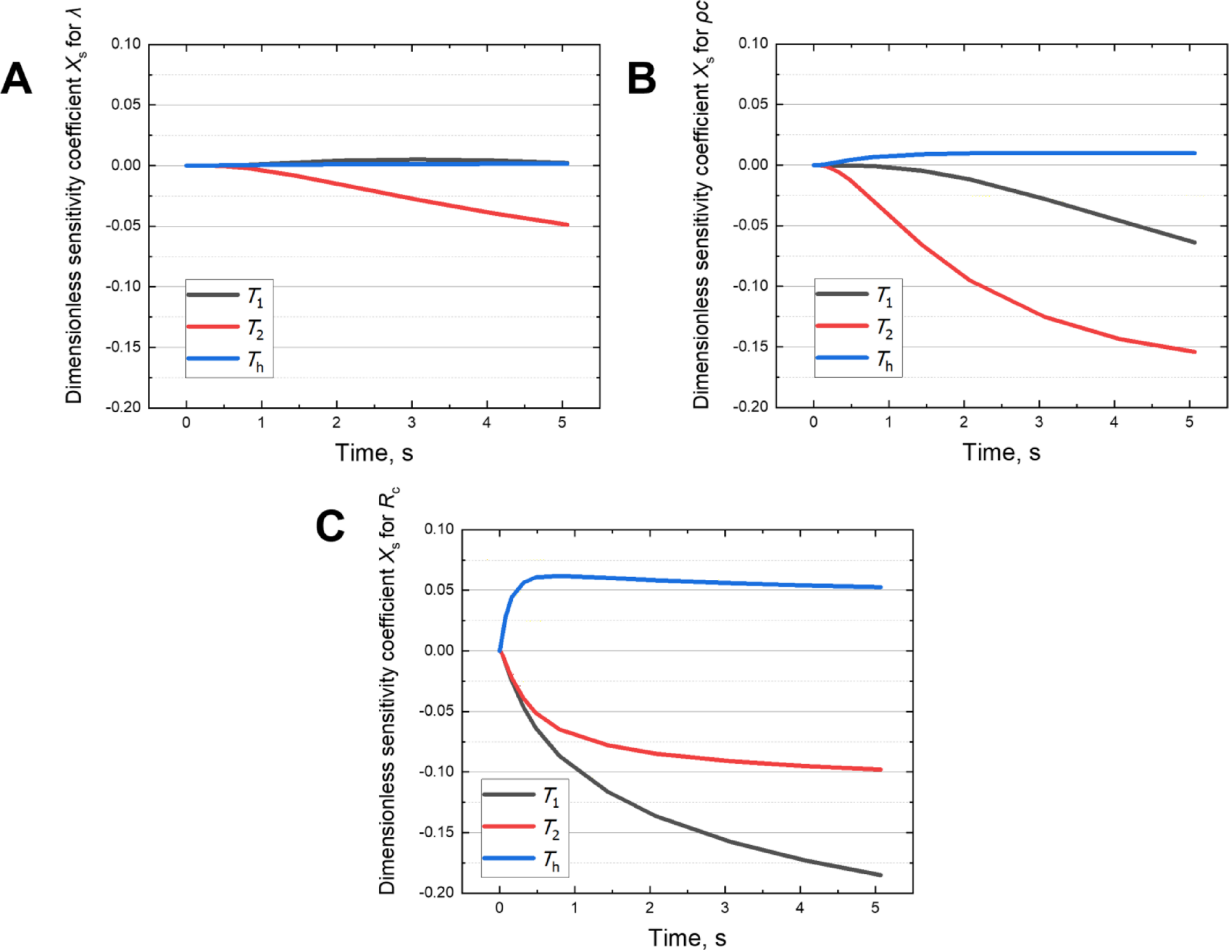
Time evolution of dimensionless sensitivity coefficients *X*_s_ with respect to the estimated parameters (**A**) thermal conductivity (*λ*), (**B**) volumetric heat capacity (*ρc*), and (**C**) thermal contact resistance (*R*_c_), based on silicone rubber data. Each panel shows the sensitivity coefficients at three temperature measurement points (*T*_1_, *T*_2_, and *T*_h_), indicating the degree to which each parameter influences the transient temperature response over time.

As shown in Fig. 7, the thermal conductivity (*λ*) is primarily identifiable through the *T*_2_ sensor, particularly during the later stages of the transient response. The volumetric heat capacity (*ρc*) also shows strong sensitivity at *T*_2_, as this location captures the delayed temperature response associated with thermal energy accumulation within the sample. In contrast, the thermal contact resistance (*R*_c_) exhibits high sensitivity at *T*_1_ during the early stage of heating, when the temperature rise is strongly affected by the thermal interface between the sensor and the sample. These findings indicate that *R*_c_ predominantly affects the initial interfacial heat transfer, whereas *λ* and *ρc* govern the subsequent heat conduction behavior within the sample.

While not exhibiting the highest sensitivity to any single parameter, the heater temperature *T*_h_ provides consistent supplementary information across all estimations. It maintains moderate sensitivity to all three parameters throughout the measurement period, thereby serving as a stabilizing reference and enhancing the robustness of the inverse analysis.

Collectively, these complementary sensitivity patterns reveal a functional differentiation among the three measurement locations. Each sensor captures distinct aspects of the thermal response, and their combined use supports accurate and stable estimation of the thermal transport properties.

Additionally, Supplementary Figure S1 shows the time evolution of the dimensionless sensitivity coefficient curves at each temperature measurement point. These complementary sensitivity profiles support the rational design of the sensor layout and demonstrate that spatial diversity in thermal responses enhances the robustness of the inverse analysis. The combined use of spatially distributed measurements enables reliable parameter estimation, particularly under the constraints of non-invasive surface sensing.

### Dependency of initial guesses

In general, gradient-based methods, including second-order approximation methods, such as the Gauss–Newton and Levenberg–Marquardt methods, are sensitive to the choice of initial values^3,24^. Different initial guesses may cause the optimization process to converge to different solutions, including local minima, and therefore require careful selection. By contrast, the trust-region reflective algorithm used in this study is known for its robustness because it can adaptively adjust the search region (trust region) even when the initial guess is poor. This allows the method to take appropriately sized steps toward the optimal solution and increases the likelihood of converging to an appropriate result.

To verify the robustness of the optimization procedure, the objective function and estimated parameters were monitored using different initial estimates (Cases 1–4), as listed in Table 2. The initial values were selected to reflect those close to the thermal transport properties of biological tissues and were deliberately varied over a wide range, which is typical for such materials.

**Table 2:** Four sets of initial guesses (Cases 1, 2, 3, and 4) for the inverse analysis with three unknown parameters.

	Thermal conductivity *λ* [W/(m·K)]	Volumetric heat capacity *ρc* [MJ/(m^3^·K)]	Thermal contact resistance *R*_c_ [× 10^−4^ (m^2^·K)/W]
Case 1 (default)	0.10	1.00	0.5
Case 2	0.20	2.00	4.0
Case 3	0.30	3.00	6.0
Case 4	0.40	4.00	8.0

**Fig. 8:**
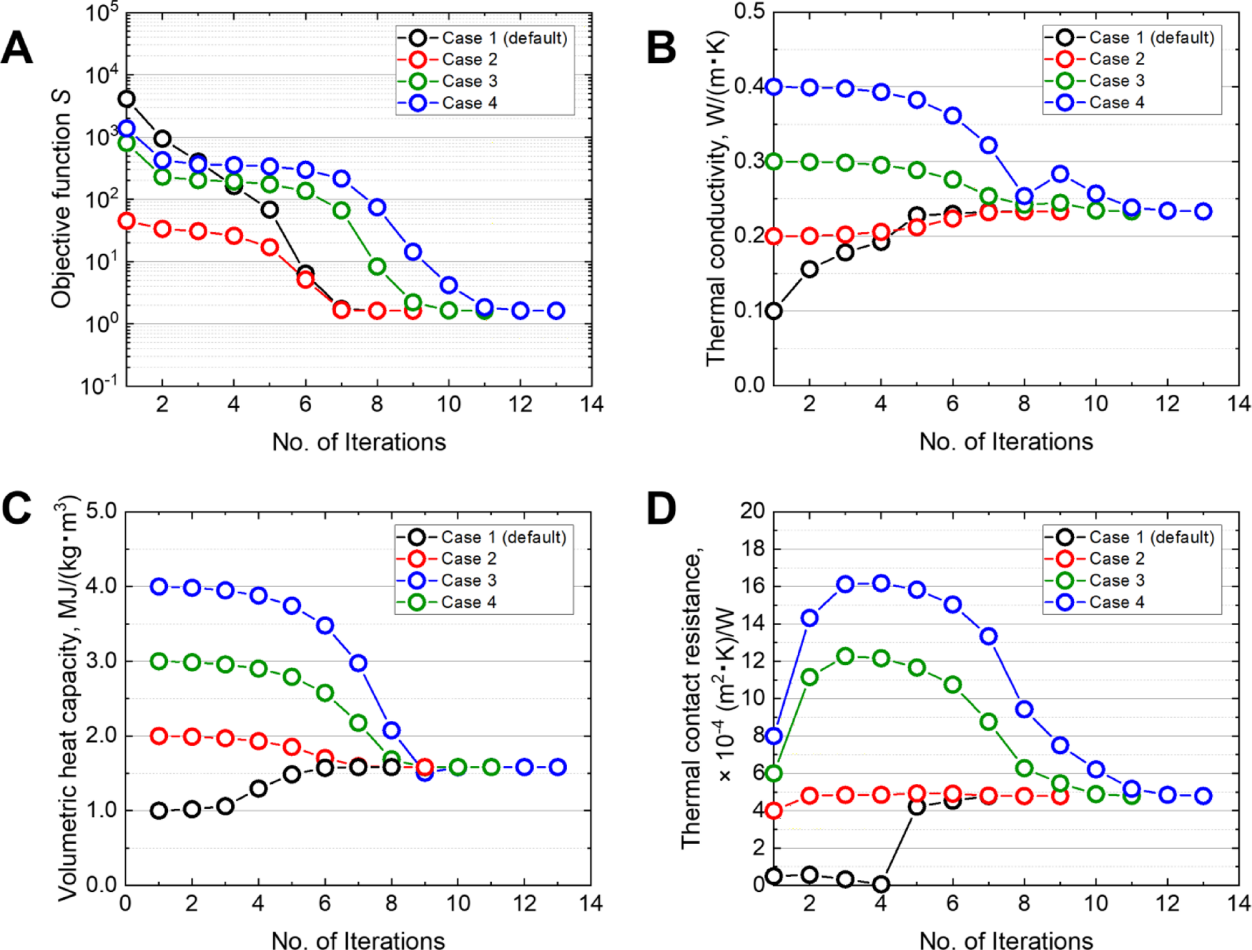
Evolution of (**A**) the objective function, (**B**) thermal conductivity, (**C**) volumetric heat capacity, and (**D**) thermal contact resistance as a function of the iteration number using different initial guesses for the inverse analysis with unknown parameters *λ*, *ρc*, and *R*_c_, for Cases 1–4 in Table 2, based on silicone rubber data.

Figure 8 shows the evolution of the objective function, thermal conductivity, volumetric heat capacity, and thermal contact resistance with respect to the iteration number for Cases 1–4 in Table 2, based on the silicone rubber data. The results indicate that although the number of iterations increased with the distance between the initial estimate and the final solution (ranging from 9 to 13 iterations), all cases consistently converged to the same solution. This trend was observed across the objective function and all estimated parameters. Moreover, a similar convergence behavior was confirmed not only for silicone rubber but also for all the other samples examined. These results suggest that, as long as the initial estimate is reasonably close to the thermal properties of the biological tissues, the proposed method can robustly and reliably converge to an accurate solution.

## Discussion

Conventional methods for measuring the thermal transport properties of biological tissues often involve invasive procedures^20,21^, long measurement durations^26^, and strict limitations on the sample geometry and measurement location^26,32,33^. Consequently, practical methods suitable for real biological applications remain scarce, and their implementation in clinical settings is extremely limited. Moreover, although surface characteristics and contact conditions can vary significantly based on the individual, tissue state, and anatomical site, few methods consider such variations in the measurement process.

By contrast, the flexible thermal sensor proposed in this study enables noninvasive, rapid, and high-sensitivity estimation of thermal transport properties while considering variations in surface conditions and contact resistance. Simply attaching the sensor to a biological surface enables reliable measurements without the need for complex preparation. Furthermore, by periodically applying the heating protocol, dynamic changes in the tissue conditions can be monitored over time. These features highlight the strong potential of the proposed method for application in medical diagnostics and other healthcare technologies. For example, it may be applied to the diagnosis of skin diseases that locally alter thermal properties, such as burns or inflammation, and for the intraoperative monitoring of tissue perfusion or viability during minimally invasive surgeries. The ability to perform localized, surface-based thermal assessments in a simple and reproducible manner can contribute to the advancement of personalized and adaptive medical care.

Additionally, the design flexibility of the proposed sensor allows it to adapt to various anatomical sites by altering its size and shape. This not only enables measurement in anatomically complex or restricted areas but also facilitates the optimization of sensor performance for specific applications. By tuning the sensor dimensions, key parameters such as the magnitude and rate of temperature rise, as well as the sensitivity to thermal property variations, can be controlled. This structural versatility is essential for tailoring sensors for a wide range of biomedical applications. Moreover, when combined with miniaturized electronics and wireless data transmission technologies, the sensor platform has strong potential for integration into wearable devices. Such integration could enable the continuous and noninvasive monitoring of tissue thermal properties in daily life, rehabilitation, or long-term healthcare, thereby expanding the use of thermal analysis from laboratory-based research to real-world personalized medicine.

In this study, an inverse analysis was performed using only two of the three thermistors, along with the heater temperature in the sensor module. This method is justified by the radial symmetry of heat conduction around the circular heater, which allows for reliable parameter estimation without requiring data from all the thermistors. However, deviations from this symmetry can provide valuable information regarding nonuniform heat transport, such as that caused by subsurface blood flow or spatially heterogeneous boundary conditions. Previous studies have explored the use of asymmetric temperature fields to estimate physiological parameters such as blood flow or local sweat secretion individually^34,35^. In contrast, the proposed sensor system has the potential to enable simultaneous estimation of thermal transport properties and physiological responses, such as blood flow and local sweat secretion, by intentionally utilizing thermal asymmetry in future implementations. This dual capability could enhance the diagnostic value of the system and open up new opportunities for real-time physiological monitoring under dynamic and variable conditions.

Thus, although the current method leverages symmetry for robustness and simplicity, the intentional use of asymmetry may extend the functionality of the sensor platform to advanced biomedical applications, including the detection of local hemodynamic changes or changes in local sweat secretion alongside intrinsic thermal characterization.

## Electronic supplementary material

Below is the link to the electronic supplementary material.

Supplementary Material 1

## Data Availability

The datasets generated and/or analyzed in the current study are available from the corresponding author upon reasonable request.
