# Supplementary material for: Flexible thin-film thermal sensor for estimating thermal transport properties designed for biomaterial applications
Source: Sci Rep. 2025 May 28;15:18648. doi: 10.1038/s41598-025-03304-0 (PMC12119917; doi:10.1038/s41598-025-03304-0)
Supplement: Supplementary file 1 — Supplementary Material 1 [file 41598_2025_3304_MOESM1_ESM.pdf]

Supplementary Information

Flexible thin-film thermal sensor for estimating thermal transport properties designed for biomaterial applications

Takahiro Okabe[1, *], Ayumi Shiroto[1], Yuto Hiyama[2], Katsuhisa Taguchi[2]

[1]Graduate School of Science and Technology, Hirosaki University, Japan.
[2]SEMITEC Corporation, Japan.
[*] Corresponding author: Takahiro Okabe

Corresponding author:

Takahiro Okabe, Ph.D. (Eng.)
Graduate School of Science and Technology
Hirosaki University, 3 Bunkyo-cho, Hirosaki, Aomori, 036-8561, Japan
Tel: +81-172-39-3615
E-mail: oka@hirosaki-u.ac.jp

Section S1. Uncertainty analysis for temperature measurement

Since the inverse analysis is based on the temperature rise at each sensor location from the initial state (i.e., the increase in temperature caused by the heater at each measurement point), rather than on absolute temperature values, systematic errors—such as those arising from sensor calibration or digital multimeter accuracy—are expected to cancel out. Although each sensor was connected to a separate digital multimeter, all resistance measurements were converted to temperature using the same calibration procedure, ensuring that any systematic bias would affect all sensors uniformly and have minimal impact on the evaluation of temperature rise. As a result, only random uncertainties due to measurement repeatability and instrument resolution were considered in the uncertainty analysis. The standard uncertainty associated with each temperature measurement point (T_1, T_2, T_h) was evaluated by combining the standard deviation obtained from repeated measurements and the resolution-related uncertainty of the digital multimeter (7352A, ADCMT). The combined standard uncertainty u_T was calculated using the following equation:

$$u_T = \sqrt{u_{\text{repeat}}^2 + u_{\text{resol}}^2}, \tag{S1}$$

where u_{repeat} is the standard deviation of repeated measurements, and u_{resol} represents the uncertainty due to instrument's resolution. The resulting combined standard uncertainties u_T and the expanded uncertainty U_T (with a coverage factor $k = 2$) are summarized in Table S1.

Notably, the expanded uncertainty for T_h was larger than those for T_1 and T_2. This difference is primarily attributed to the sensor characteristics: the heater signal was derived from a copper foil with a low resistance (≈ 150–185 Ω), resulting in a smaller resistance change per degree and lower sensitivity to temperature variation. In contrast, the NTC thermistors (T_1 and T_2) feature higher temperature sensitivity and signal magnitude, leading to smaller measurement fluctuations and higher precision. These differences in sensor behavior reasonably account for the variation in uncertainty values.

Table S1. Combined standard uncertainties u_T and expanded uncertainty U_T (coverage factor $k = 2$) for each temperature measurement point (T_1, T_2, T_h), calculated based on measurement repeatability and digital multimeter resolution.

Measurement point	u_{repeat} [K]	u_{resol} [K]	Combined standard uncertainty u_T [K]	Expanded uncertainty U_T [K] ($k = 2$)
T_1	0.0082	0.0007	0.0082	0.0164
T_2	0.0135	0.0007	0.0135	0.0269
T_h	0.0588	0.0043	0.0599	0.1179

Section S2. Uncertainty analysis for inverse analysis

To evaluate how experimental uncertainties affect the estimated thermal parameters, an uncertainty analysis based on the Monte Carlo Method (MCM) was performed. This approach was adopted because the inverse problem is inherently nonlinear and cannot be addressed analytically using the conventional GUM framework. Accordingly, the MCM was employed to numerically propagate uncertainty through the parameter estimation process [S1].

Random noise was added to the original temperature signals to evaluate how measurement uncertainty propagates through the inverse analysis. Thirty synthetic datasets were generated by adding normally distributed noise —based on the combined standard uncertainties listed in Table S1— to the original temperature data at each measurement point (T_1, T_2, and T_h). The inverse analysis was independently conducted to estimate the thermal conductivity λ, volumetric heat capacity ρc, and thermal contact resistance R_c.

The resulting distributions of the estimated parameters reflect the propagated effects of measurement uncertainty through the nonlinear inverse analysis. Table S2 summarizes the mean, standard deviation (1σ), minimum, maximum, and expanded uncertainty (2σ) for each parameter. These results confirm the robustness and stability of the proposed method under realistic experimental conditions. This simulation-based uncertainty quantification provides a practical and reliable estimate of confidence intervals for the identified thermal transport parameters.

Table S2. Summary of thermal parameters estimated from 30 Monte Carlo trials using perturbed temperature datasets. Expanded uncertainty U is reported with a coverage factor $k = 2$ (based on silicone rubber data).

Parameter	Mean	Standard deviation (1σ)	Min	Max	Expanded uncertainty U (2σ)
λ [W/(m·K)]	0.233	0.002	0.23	0.236	0.003 (1.3%)
ρc [MJ/(m^3·K)]	1.583	0.01	1.565	1.609	0.021 (1.3%)
R_c [$\times 10^{-4}$ (m^2·K)/W]	4.789	0.088	4.609	4.96	0.176 (3.7%)

[S1] JCGM 101:2008. Evaluation of measurement data — Supplement 1 to the “Guide to the expression of uncertainty in measurement” — Propagation of distributions using a Monte Carlo method. Joint Committee for Guides in Metrology (2008).

Section S3. Sensitivity analysis

Figure S1 shows the time evolution of dimensionless sensitivity coefficients (X_s) at each temperature measurement point. This analysis complements the inverse analysis presented in the main text (Fig. 7) and demonstrates how different sensor positions (T_1, T_2, T_h) contribute uniquely to the identifiability of the three estimated thermal parameters. Such diversity supports the robustness and stability of the parameter estimation process.

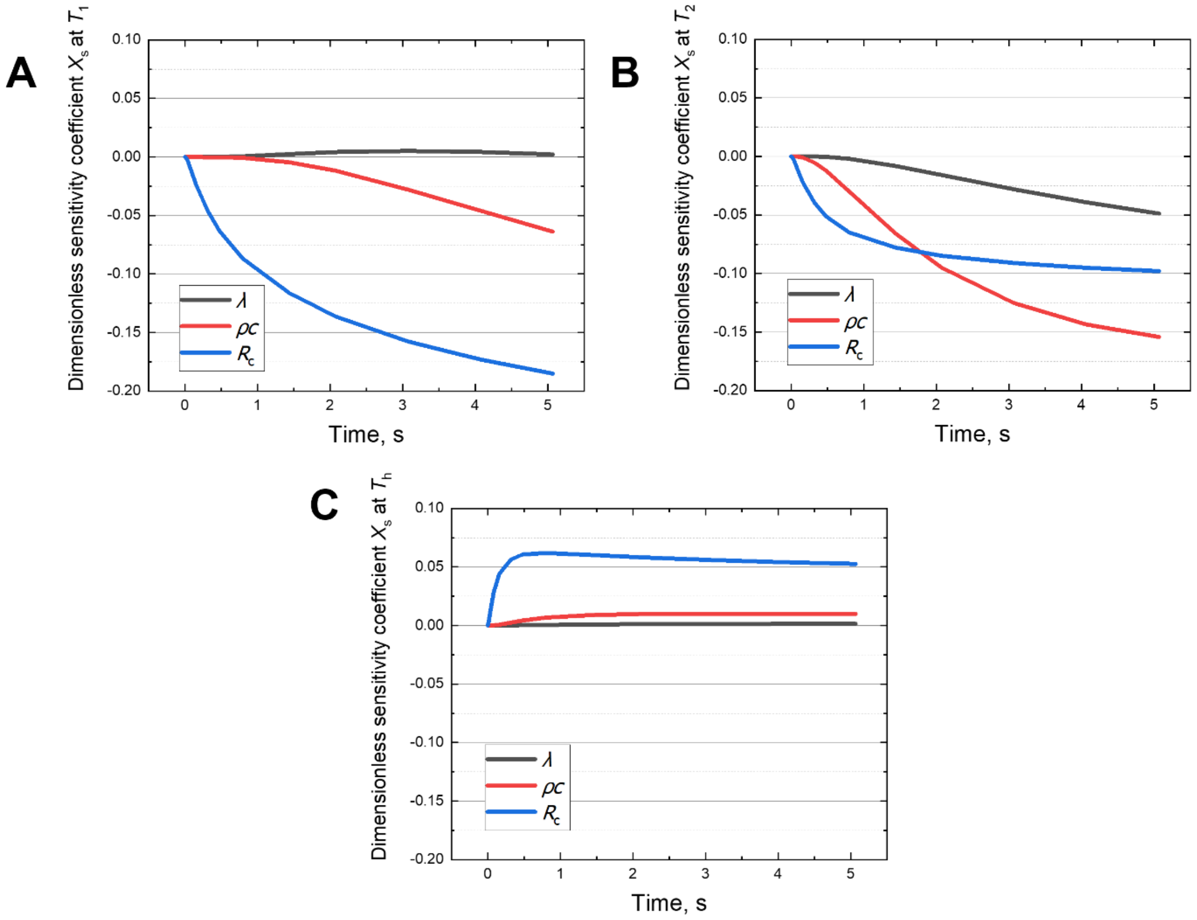

Figure S1. Time evolution of dimensionless sensitivity coefficients (X_s) at each temperature measurement point: (A) T_1, (B) T_2, and (C) T_h. Each curve represents the sensitivity (X_s) with respect to one of the three estimated parameters: thermal conductivity (λ), volumetric heat capacity (ρc), and thermal contact resistance (R_c).

Section S4. Simulated temperature distributions in the sensor-sample system

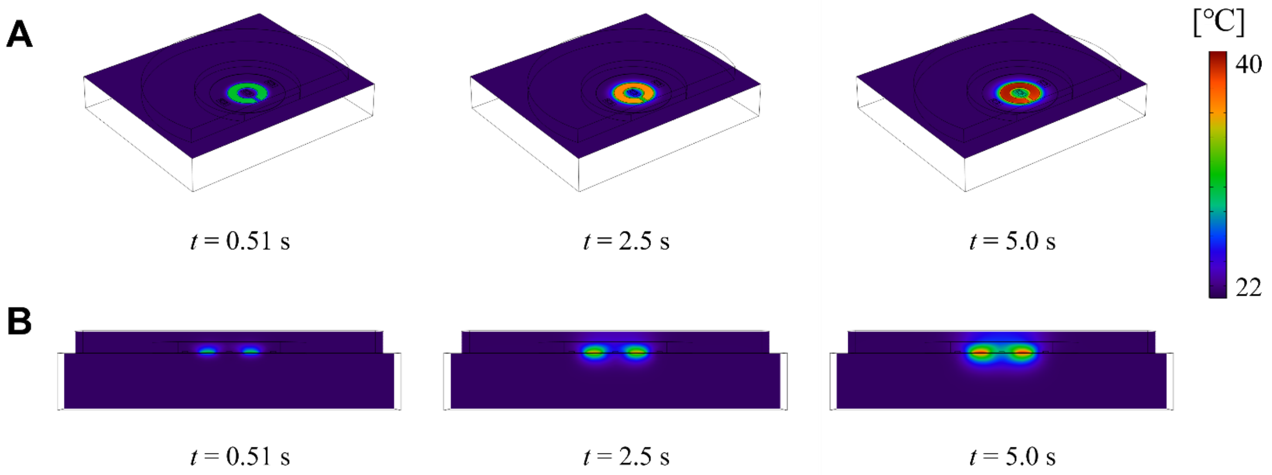

Figure S2. Simulated temperature distributions in the sensor-sample system at three representative time points (t = 0.51 s, 2.5 s, and 5.0 s). (A) *xy*-plane surface views showing lateral heat spread at the sensor-sample interface. (B) *xz*-plane cross-sections at the sensor center illustrating vertical heat penetration into the sample.
